# Case 6 / 2018 - Percutaneous Occlusion of a Large Ductus Arteriosus in a Low Weight Infant, with Immediate Clinical and Radiographic Improvement

**DOI:** 10.5935/abc.20180219

**Published:** 2018-11

**Authors:** Pablo Tomé Teixeirense, Vanessa de Moraes Sousa, João Felipe Barros de Toledo, Luiz Antonio Gubolino

**Affiliations:** Irmandade de Santa Casa de Misericórdia de Limeira, Limeira, SP - Brazil

**Keywords:** Infant, Down Syndrome, Heart Defects, Congenital/surgery, Ductus Arteriosus Patent/surgery

## Clinical Data

The patient was a one-year-old infant with Down syndrome, and heart murmur auscultated from birth. The child had a difficult clinical course due to failure to thrive, tachypnea, poor suckling due to fatigue and repeated respiratory infections, with pulmonary hypersecretion, and was receiving captopril and furosemide.

### Physical examination

Regular overall status, tachypneic, acyanotic, with full and wide peripheral pulses. Weight: 8.6 kg, height: 71 cm, blood pressure in the right upper limb: 80 x 40 mmHg, HR: 148 bpm, O_2_Sat: 97%. The apex beat was shifted to the left in the precordium, in clear systolic impulse. Continuous "machine-like” murmur, better auscultated at the left sternal border and irradiating to the posterior chest region. Palpable liver two centimeters from the right costal ridge and diffuse rumbles and subcrepitant rales at the lung bases.

### Complementary examinations

**Electrocardiogram:** sinus rhythm (tachycardic), with left shift and left ventricular overload.

**Figure 1:**
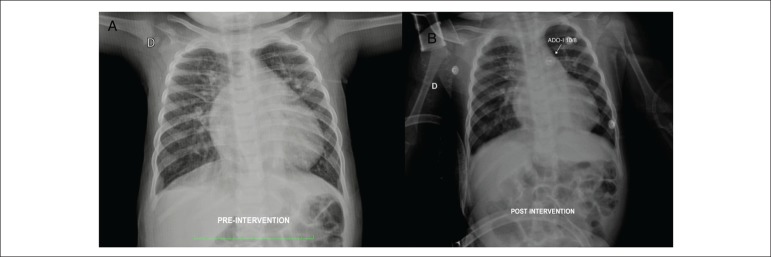
A) Pre-intervention chest x-ray. There is an overall increase in the cardiac silhouette, with prominence of the right atrium, left ventricle and vascular pedicle, in addition to the pulmonary vascular network. B) Chest X-ray approximately 8h after occlusion of the defect, showing the significant decrease in the cardiac volume, notably in the right atrium and the vascular pedicle, as well as a decrease in the pulmonary vascular network

**Chet x-ray:** enlarged cardiac area with a cardiothoracic index of 0.64, marked vascular pedicle enlargement, and increased pulmonary vascular network (Figure 1A).

**Echocardiogram:** enlargement of the left chambers, significant dilatation of the pulmonary trunk and pulmonary arteries, and presence of a ductus arteriosus with left-to-right shunt, with the smallest diameter estimated at 4 mm.

### Clinical diagnosis

Patent ductus arteriosus with significant hemodynamic consequences in an infant with Down syndrome.

### Differential diagnosis

Other congenital defects should always be recalled in a similar clinical setting such as: defects between the systemic and pulmonary sites, the aortopulmonary window that connects the ascending aorta and the pulmonary trunk, coronary-cavitary fistulas and arteriovenous defects in general, total anomalous pulmonary vein drainage, sinus of Valsalva rupture, and pulmonary atresia with enlarged bronchial arteries or large systemic-pulmonary collateral vessels, which allow pulmonary flow increase.

### Conduct

**Figure 2:**
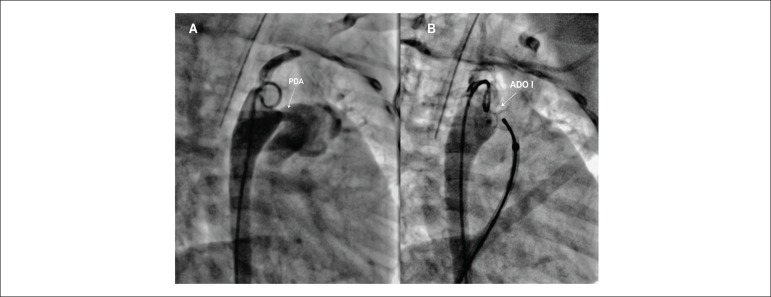
A) Angiography of the aorta showing the presence of a large ductus arteriosus with a minimum diameter of 4 mm. B) Implant of Amplatzer® device ADO I-10/8, with complete occlusion of the defect. PDA: patent ductus arteriosus

Due to the infant’s clinical impact and failure to thrive, the first considered conduct was percutaneous occlusion through interventional catheterization techniques. The procedure was performed through femoral vein and artery puncture, with hemostasis valve 4F to minimize the risk of peripheral vascular lesions. Manometric study disclosed marked pulmonary hypertension (PT = 45/25 mmHg), corresponding to half of the systemic pressure. The left ventricle showed increased end-diastolic volume, but with preserved contractile function. The aortic arch was shifted to the left and there was a large ductus arteriosus (Figure 2A), type A, according to Krichenko classification, with pulmonary extremity measuring 4.0 mm and aortic 8.0 mm, with a very prominent aortic ampulla, measuring 12 mm in diameter. In this case, we chose to use an Amplatzer® ADO-I 10/8 device with complete occlusion of the defect after its implantation (Figure 2B).

The clinical improvement was immediate with disappearance of the continuous murmur, normal breathing and obvious respiratory relief. The chest radiography, approximately 8 hours after the procedure, showed a marked decrease in the cardiac area with a cardiothoracic index of 0.58 (Figure 1B). The patient was discharged after 48 hours of hospitalization.

### Comments

After the percutaneous closure of the ductus arteriosus, a marked decrease in pulmonary hyperflow was observed immediately, due to the decreased cardiac volume and smaller vascular pedicle, as shown by the chest X-ray (Figure 1B). Before that, a marked volume overload was observed on the heart and the hemodynamic consequences to the patient with dyspnea and delayed physical development, consequent to the large ductus arteriosus.

It is concluded that the patent ductus arteriosus occlusion should be performed as soon as possible in this clinical situation, considering the several complications that may affect patient evolution, such as frequent respiratory infections, as well as the progression of pulmonary arterial hypertension to Eisenmenger's syndrome.

The occlusion techniques through interventional catheterization are safe and simple, and with catheter profile improvement and the multiple devices available for clinical use, they are currently the first choice techniques for the treatment of young infants and children.^1^ Several articles have been published on the experience of several groups showing the practice of occlusion of ductus arteriosus in extremely preterm infants,^2^^,^^3^ using only venous access and monitoring the implant through echocardiography, thus reserving the surgical technique for special anatomical situations.
